# Comprehensive analysis of serum cytokines in patients with multiple myeloma before and after lenalidomide and dexamethasone

**DOI:** 10.1002/cam4.70019

**Published:** 2024-07-19

**Authors:** Takuto Tachita, Masaki Ri, Sho Aoki, Arisa Asano, Takashi Kanamori, Haruhito Totani, Shiori Kinoshita, Yu Asao, Tomoko Narita, Ayako Masaki, Asahi Ito, Shigeru Kusumoto, Hirokazu Komatsu, Shinsuke Iida

**Affiliations:** ^1^ Department of Hematology and Oncology Nagoya City University Graduate School of Medical Sciences Nagoya Japan; ^2^ Department of Gastroenterology and Hematology Hirosaki University Graduate School of Medicine Hirosaki Japan

**Keywords:** IL‐18, lenalidomide, M‐CSF, multiple myeloma, PDGF‐BB

## Abstract

Multiple myeloma (MM) is an incurable B‐cell malignancy often accompanied by profound immunodeficiency. Lenalidomide (Len) is an immunomodulatory drug that exerts promising therapeutic effects on MM through the immune system. However, predictive markers related to the effects of Len treatment are not fully understood. This study aimed to identify candidate biomarkers for predicting the clinical efficacy of Len and dexamethasone (Ld) therapy through a comprehensive analysis of serum cytokines. The levels of 48 cytokines in the serum of patients with MM just before Ld therapy (*n* = 77), at the time of best response (*n* = 56), and at disease progression (*n* = 49) were measured and evaluated. Patients with high IL‐18 and M‐CSF levels showed significantly shorter progression‐free survival and overall survival (OS). In contrast, patients with high PDGF‐BB levels had longer survival. Moreover, low levels of G‐CSF, IL‐7, IL‐8, and SDF‐1α were associated with shorter OS after Ld therapy. During Ld therapy, pro‐inflammatory cytokines such as IL‐2Rα, IL‐18, and TNF‐α were decreased, while IFN‐γ was increased. IL‐4 and IL‐6 levels increased during disease progression. In conclusion, this study provides a better understanding of the association between cytokines and the efficacy of Ld therapy as well as the unique changes in cytokines related to inflammatory and immune responses during Ld therapy.

## Graphical abstract

We investigated the prognostic value of the comprehensive analysis of serum cytokines in patients with multiple myeloma treated with lenalidomide and dexamethasone therapy. Patients with high IL‐18 levels and high M‐CSF levels showed a significantly shorter progression‐free survival and overall survival. On the other hand, patients with low PDGF‐BB levels showed shorter survival.

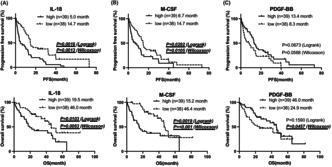

## 1 INTRODUCTION

The systemic cytokine environment plays a pivotal role in the growth and progression of hematological malignancies. In lymphoid malignancies, cytokines play a role in tumor survival and tumor microenvironment. For example, in our previous report, high levels of IL‐5 and IL‐10 were unfavorable prognostic factors in adult T‐cell leukemia/lymphoma.^1^ Classical Hodgkin lymphoma is characterized by the overexpression of Th2 cytokines and chemokines and suppression of the Th1 cell‐mediated cellular immune response.^2^ Several studies have been conducted to clarify the profile and pathophysiological implications of cytokines in multiple myeloma (MM). Zheng et al. evaluated the cytokine profile of patients with MM and reported an anti‐inflammatory phenotype of multiple cytokines that support tumor growth by escaping from immunosurveillance.^3^ Saltarella et al. reported the presence of proangiogenic cytokines in the bone marrow milieu of patients with MM. They have also shown that FGF‐2, HGF, VEGF, and PDGF‐β levels have predictive significance for response to MM treatment.^4^ To date, the relationship between each cytokine and the response to specific therapies has been poorly investigated.

Lenalidomide (Len) is an immunomodulatory drug (IMiD) that plays an important role in the treatment of MM. This drug is often used in combination with dexamethasone and is also known as Ld therapy. Len enhances the host's antitumor immune response; thus, a combination with monoclonal antibodies such as elotuzumab^5^ and daratumumab^6^, ^7^ is considered an efficient treatment option for relapsed/refractory (RR) cases of MM. In a previous report, we investigated the prognostic value of the expression of CRBN pathway genes on the clinical relevance of Len treatment and demonstrated the alteration of these genes, reduced expression of *IKZF1* and increased expression of *KPNA2*, as possible biomarkers for the prediction of poor outcomes in Ld therapy.^8^ However, as a limitation, this study mainly investigated the antitumor effect of Len on MM cells and did not fully investigate the effect of Len on the tumor microenvironment supporting tumor survival during continuous Len treatment.

Little is known about the alteration of the tumor microenvironment and immune function in patients undergoing prolonged Ld therapy. Systemic cytokine profiles are expected to explore the environmental factors highly associated with the tumor microenvironment and immune factors targeting MM cells; however, these profiles are poorly understood in terms of Ld therapy.^9^ In this study, we aimed to elucidate the changes in cytokines induced by Ld therapy and identify the association of several cytokines with the clinical efficacy of Ld therapy through a comprehensive analysis of serum cytokines.

## 2 MATERIALS AND METHODS

### 2.1 Clinical data and preparation of samples from patients with RR MM

We retrospectively analyzed 77 patients with RR MM who received Ld therapy between 2007 and 2018 at the Nagoya City University Hospital (Aichi, Japan). Serum samples were obtained from the 77 patients immediately before Ld therapy (pre‐Ld). Later, serum samples were collected from 56 patients at the point of treatment response (best response (BR)) and 49 patients at disease progression (PD) after Ld therapy. We performed a longitudinal analysis in as many patients as possible. Peripheral blood samples were obtained from all group (pre‐Ld, BR, and PD) in the Day 1 of Ld therapy. All patients provided written informed consent prior to peripheral blood sampling according to the requirements of the Declaration of Helsinki.

All the patients were assessed using the International Staging System of the International Myeloma Working Group (IMWG).^10^ The response to therapy was evaluated using the uniform response criteria of the IMWG. The timing of best response was defined as the best response during Ld therapy in patients who had an event (relapse or death). On the other hand, patients who did not have an event were censored at the date of last observation. Their best response was defined as the best response from the initiation of Ld therapy to the date of last observation. Progression‐free survival (PFS) was defined as the time from the initiation of Ld therapy to disease progression or death from any cause. Overall survival (OS) was calculated from the initiation of therapy until death from any cause. Using global RT‐PCR of bone marrow samples, the expression of three translocation‐related genes (*CCND1*, *FGFR3*, and *c‐MAF*) was analyzed in primary MM cells as described previously.^11^

### 2.2 Sample preparation

Peripheral blood samples were transferred to serum‐separating tubes and centrifuged at 3000 rpm at 20°C for 10 min after clot formation. Supernatants were carefully harvested, and aliquots were frozen at −80°C until analysis.

### 2.3 Cytokine measurement by Bio‐Plex multiplex system

Bio‐Plex Pro Human Cytokine Assays (Bio‐Rad, USA) were used to quantify 48 cytokines (Table S1). Serum samples were diluted fourfold (1:4) by adding 12.5 μL serum and 37.5 μL sample diluent. The assay was performed according to the manufacturer's instructions. Bead fluorescence readings were analyzed using a Bio‐Plex MAGPIX multiplex reader (Bio‐Rad, USA), and cytokine levels were determined using Bio‐Plex Manager Software (Bio‐Rad, USA) in duplicate samples. Cytokines for which most data were outside the range of the standard curve were excluded from further analysis.

### 2.4 Statistical analysis

Statistical analyses were performed using GraphPad Prism software (version 8; GraphPad Software, San Diego, CA, USA). Statistical significance was set at *p* < 0.05. The median expression levels of cytokines were compared using Mann–Whitney *U*‐ and Kruskal‐Wallis tests. Best response between the two groups of each cytokine compared by Fisher's exact test. Survival was compared using the Kaplan–Meier method with log‐rank and Wilcoxon tests. Univariate and multivariate analysis for survival with the Cox proportional hazard model.

## 3 RESULTS

### 3.1 Patient characteristics

Patient characteristics are described in Table 1. Seventy‐seven patients received Ld therapy. The median age and median number of prior therapies were 69 years and 2, respectively. One (1%), 11 (14%), 42 (55%), and 18 (23%) patients achieved complete response (CR), very good partial response (VGPR), partial response (PR), and stable disease (SD), respectively. Sixty‐three patients (82%) had previously received bortezomib‐containing therapies, and 17 patients (22%) had previously received thalidomide‐containing therapies.

**TABLE 1:** Patient characteristics.

Number of patients	77	
Sex		
Male/female	31/46	
Age (year)		
Median (range)	69 (44–84)	
M‐protein		
IgG	41	53%
IgA	15	19%
BJP	17	22%
IgD	3	4%
Non secretory	1	1%
ISS stage at diagnosis		
Stage 1	18	23%
Stage 2	31	40%
Stage 3	27	35%
ND	1	1%
RT‐PCR and fish		
*CCND1*	32	42%
t(11;14)	17	22%
11 polysomy	4	5%
NA	7	9%
ND	4	5%
*FGFR3*	17	22%
t(4;14)+	11	14%
t(14;16)+	2	3%
NA	4	8%
*c‐MAF*	16	21%
t(14;16)+	6	8%
NA	9	12%
ND	1	2%
Triple negative^a^	13	17%
t(4;14)+	3	4%
ND	12	16%
ASCT		
+/–	25/52	
Prior therapies		
Median (range)	2 (1–6)	
Prior bortezomib therapy		
+/–	63 (82%)/14	
Prior thalidomide therapy		
+/–	17 (22%)/60	
Best response		
CR	1	1%
VGPR	11	14%
PR	42	55%
MR	1	1%
SD	18	23%
PD	4	5%
Timepoint of sampling		
Just before of Ld therapy	77	
Best response of Ld therapy	56	
Progression of disease	49	

Abbreviations: CR, complete response; MR minimal response; NA, no abnormality; ND, not done; PD, progressive disease; PR, partial response; SD, stable disease; VGPR, very good partial response.

^a^ *CCND1‐*, *FGFR3‐*, *c‐MAF‐* negative in RT‐PCR.

In Table S2, the initial dose of Ld therapy was summarized. Patients had received Ld therapy for a median of 240 days (range: 3–1685 days).

We compared cytokine levels between each ISS stage. Consequentially, IL‐2Ra and MIG were significantly associated with the ISS stage (Kruskal–Wallis test, *p*‐value <0.05) (Figure S2). We compared each cytokine levels between the group with prior ASCT and with no prior ASCT. There was no significant difference between the two groups (data not shown). Figure S3 displays the association pre‐Ld cytokine levels and the previous treatment. Cytokine levels of CTACK, Eotaxin, IL‐1ra, IL‐4, IL‐8, IL‐17A, MIF, RANTES, SDF‐1α, TNF‐α, and TRAIL were lower in the patients who had prior Bor therapy. On the other hand, serum IL‐10 levels were higher in the patients who had prior Thal therapy. This result was consistent with the previous report suggested that Thal enhanced IL‐10.^12^ We compared serum cytokine levels between the two groups based on chromosomal abnormalities (CA) and LDH level (Figure S4). IL‐18, M‐CSF, HGF, IL‐1α, and IL‐5 were significantly higher in high LDH group. It seems that these cytokines might reflect tumor volume of MM. The levels of β‐NGF were significantly lower in the patients with high‐risk CA included t(4; 14) and t(14; 16).

### 3.2 Impact of cytokine expression levels on response and survival after Ld therapy

**FIGURE 1:**
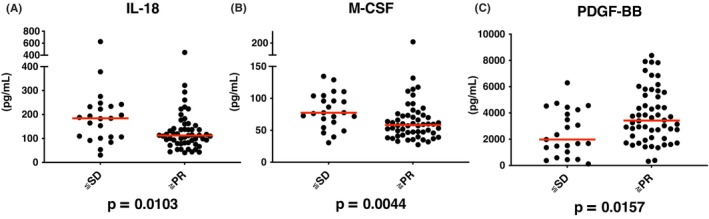
Association between pre‐Ld cytokine levels and best response after Ld therapy. Patients were classified as good responders (complete response [CR] + very good partial response [VGPR] + partial response [PR]) or poor responders (minimal response [MR] + stable disease [SD] + progressive disease [PD]). The horizontal red bars represented the median values of the levels of each group. (A, B) The expression levels of IL‐18 and M‐CSF were significantly higher in poor responders to Ld therapy. (C) The concentration of PDGF‐BB was significantly lower in poor responders.

Based on their responses to Ld therapy, patients were classified as good responders (CR + VGPR + PR, *n* = 54) or poor responders (minimal response [MR] + SD + progressive disease [PD], *n* = 23) as described previously.^8^, ^13^, ^14^ IL−18 and M‐CSF expression levels were significantly higher in poor responders to Ld therapy (*p* = 0.0103, 0.0044). In contrast, the concentration of PDGF‐BB was significantly lower in poor responders (*p* = 0.0157) (Figure 1). In Table S3, we compared the best response between the groups showing low and high levels of each cytokine by Fisher's exact test. We used median values and the values using ROC curve estimation as cutoff values, respectively. Significant differences were observed in almost groups showing low and high expression of each cytokine.

**FIGURE 2:**
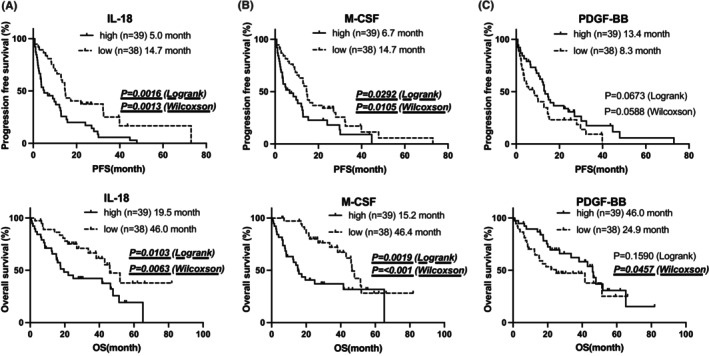
Association between pre‐Ld cytokine levels and survival after Ld therapy. (A, B) PFS and OS were significantly shorter in the group with high IL‐18 and M‐CSF levels. (C) No significant differences were observed in PDGF‐BB, but PFS and OS were shorter in the group with low PDGF‐BB levels.

All the patients were divided into two groups based on the median values of each cytokine and the level of expression (high or low), and the differences in survival were evaluated. PFS and OS were significantly shorter in the group with high IL‐18 levels (PFS, log‐rank, *p* = 0.0016; Wilcoxon, *p* = 0.0013 and OS: log‐rank, *p* = 0.0103; Wilcoxon, *p* = 0.0063) and high M‐CSF levels (PFS: log‐rank, *p* = 0.0292; Wilcoxon, *p* = 0.0105 and OS: log‐rank, *p* = 0.0019; Wilcoxon, *p* < 0.0001). Although no significant differences were observed in PDGF‐BB levels, PFS, and OS tended to be shorter in the group with low PDGF‐BB levels (Figure 2).

**FIGURE 3:**
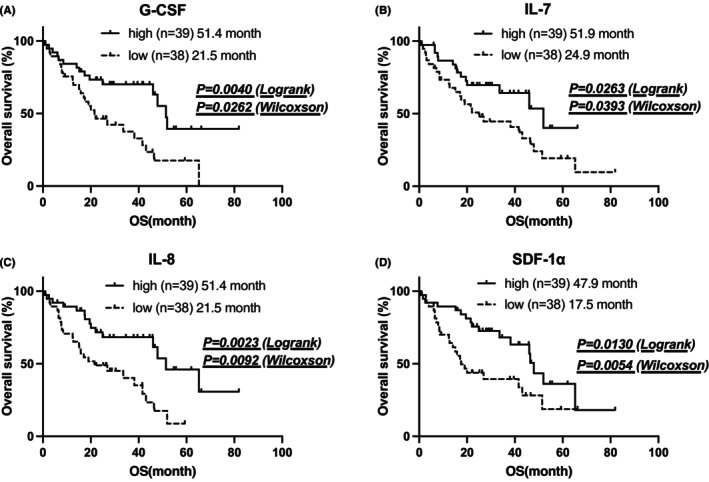
Association between pre‐Ld cytokine levels and overall survival after Ld therapy. (A–D) OS was significantly shorter in the group with low G‐CSF, IL‐7, IL‐8, and SDF‐1α levels.

When limited to OS, significant differences were observed in G‐CSF, IL‐7, IL‐8, and SDF‐1α levels. OS was significantly shorter in the group with low levels of these cytokines (Figure 3).

For progression free survival, IL‐18 levels and LDH were independent unfavorable prognostic factors. On the other hand, IL‐8 levels were independent unfavorable prognostic factors for OS. Although no statistical significance was observed, IL‐18 levels tended to be associated with shorter OS after Ld therapy (*p* = 0.053) (Tables S4 and S5).

We analyzed the correlation between IL‐18, M‐CSF, and PDGF‐BB. Using a linear regression model, the correlation between IL‐18 and M‐CSF was shown (Figure S5).

### 3.3 Alteration of cytokine expression levels before, during, and after Ld therapy

**FIGURE 4:**
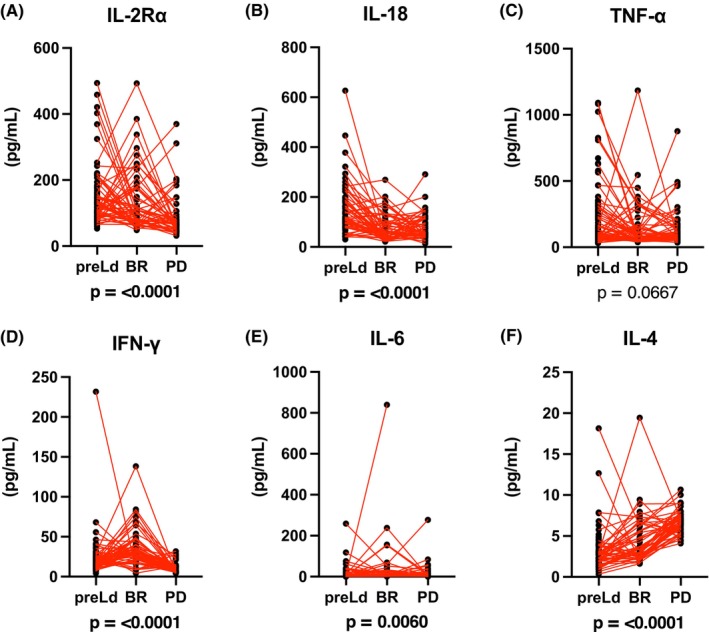
Comparison of serum cytokine levels in patients with MM treated with Ld therapy at different disease stages. (A–F) Levels of each cytokine at different disease stages (i.e. pre‐Ld therapy (pre‐Ld), at the best response (BR), and progressive disease (PD)) were shown.

Altered cytokine expression levels before, during, and after Ld therapy are shown in Table 2 and Figure S1. In this study, pro‐inflammatory cytokines such as IL‐2Rα, IL‐18, and TNF‐α were decreased by Len treatment (Figure 4A–C). In contrast, IFN‐γ was elevated by Ld therapy (Figure 4D). During PD, TNF‐α remained low, IFN‐γ was decreased, but IL‐18 was elevated. Th2 cytokines such as IL‐6 and IL‐4 were elevated in patients with PD (Figure 4E,F).

**TABLE 2:** The expression levels of serum cytokines.

Cytokine/chemokine	PreLd (pg/mL)			BR (pg/mL)			PD (pg/mL)		
	Median	Q1	Q3	Median	Q1	Q3	Median	Q1	Q3
β‐NGF^a^	1.68	0.66	3.7	1.99	1.52	2.61	5.77	2.99	9.84
CTACK	1483.28	1180.36	2064.85	955.955	737.4575	1285.5	1408.92	1124.91	1735.57
Eotaxin	94.35	73.23	124.06	89.32	70.7575	126.8325	59.37	45.14	96.55
Basic FGF	47.8	41.26	52.555	52.61	48.225	59.51	53.935	42.37	65.3125
G‐CSF	747.99	409.18	1019.12	259.44	139.6075	529.1925	198.13	99.03	443.31
GM‐CSF^a^	5.34	2.0775	12.67625	1.24	0.5125	2.4975	5.17	5.17	5.17
GRO‐α	319.81	248.69	380.81	365.795	318.93	490.425	426.025	365.775	475.825
HGF	729.64	547.26	1288.09	456.82	368.365	717.245	240.51	162.33	332.57
IFN‐α2^a^	12.815	8.7525	18.45	12.895	10.56	18.43	14.16	8.86	16.67
IFN‐γ	17.17	12.69	22.99	31.295	22.4075	41.135	7.04	5.53	12.76
IL‐1α^a^	24.62	15.3175	34.77	18.745	13.39	28.115	6.72	4.615	17.63
IL‐1β	8.5	5.37	20.15	3.34	2.91	4.43	3.46	1.065	8.24
IL‐1ra	439.49	273.68	639.67	263.87	215.31	352.55	353.305	233.5375	421.5375
IL‐2^a^	13.76	6.455	19.055	2.77	1.2575	7.4	12.18	12.18	12.18
IL‐2Rα	119.23	93.87	167.64	99.055	72.5775	155.5225	67.35	51.67	105.91
IL‐3^a^	0.35	0.21	0.58	0.23	0.15	0.69	0.83	0.83	0.83
IL‐4^a^	2.83	1.97	3.895	3.135	2.23	5.6025	6.78	5.74	7.61
IL‐5^a^	30.135	21.74	47.5875	10.6	5.9625	16.06	27.12	20.91	33.33
IL‐6^a^	3.92	1.8	7.965	3.56	1.665	14.765	7.48	2.78	18.25
IL‐7	61.02	48.28	79.57	8.83	6.52	12.625	7.75	2.54	10.04
IL‐8	205.11	65.32	636.53	27.835	14.3475	188.3175	35.18	6.8875	155.3525
IL‐9	199.01	145.14	215.945	211.32	179.7175	219.69	111.25	89.9	160.38
IL‐10^a^	9.55	6.24	13.24	5.89	4.34	10.065	4.04	1.51	7.095
IL‐12p40^a^	2.58	2.01	18.33	2.2	1.65	3.02	4.96	4.96	4.96
IL‐12p70^a^	78.87	52.065	145.415	127.88	100.86	205.43	324.62	189.77	752.69
IL‐13	8.31	5.29	14.5	1.54	0.83	2.375	3.395	1.6725	5.6725
IL‐15^a^	328.61	169	409	546.08	296.395	922.93	567.77	369.39	854.1
IL‐16	112.83	65.6	154.11	43.94	35.0725	57.9925	41.26	30.34	61.6
IL‐17A	35.29	29.77	44.44	9.81	8.565	13.65	5.51	3.84	7.2
IL‐18	117.09	92.28	184.43	48.705	33.81	91.615	66.13	47.61	114.91
IP‐10	1390.13	1000.35	1946.59	844.09	603.8325	1346.2425	643.55	447.76	880.63
LIF^a^	21.05	12.07	34.45	52.12	43.58	69.65	73.875	46.5875	107.005
M‐CSF	61.995	47.4	81.6	39.475	31.35	56.55	28.46	23.76	38.37
MCP‐1	85.58	54.99	141.12	27.535	21.03	35.545	24.81	13.02	43.48
MCP‐3^a^	2.01	1.345	3.595	0.98	0.35	2.52	1.95	1.78	2.335
MIF	1800.36	1134.56	2749.97	541.965	373.7875	849.655	705.9	399.1	1332.87
MIG	839.84	559.5	1493.06	604.46	355.5225	1239.9575	265.26	180.16	424.68
MIP‐1α^a^	146.765	44.08125	341.6475	16.8	4.6375	76.1075	43.96	6.63	144.16
MIP‐1β	240.335	127.27	495.13	198.115	185.3575	264.9725	276.31	193.67	452.45
PDGF‐BB	3068.78	1694.46	4528.42	1099.89	801.8975	1611.6875	848.52	540.41	1044.67
RANTES^a^	13,992.34	11,398.59	18,747.33	8503.415	7659.835	9973.665	7230.1	5877.99	8619.21
SCF	238.08	180.685	306.75	100.385	76.31	152.3375	105.89	77.32	152.57
SCGF‐β	240,916.33	178,435.24	323,025.83	161,024.86	134,052.91	218,885.74	109,498.44	72,553.47	141,708.78
SDF‐1α	484.13	411.48	589.7	486.14	436.905	563.08	295.22	248.44	420.45
TNF‐α	136.95	66.31	306.11	80.965	57.06	147.31	88.7	63.05	171.48
TNF‐β^a^	2.81	1.67	5.445	257.135	244.8725	269.99	143.7	114.74	235.96
TRAIL	89.55	74.18	115.62	76.345	69.59	80.2625	48.185	24.7925	71.5775
VEGF^a^	274.88	172.24	373.58	92.435	60.86	167.6825	344.21	193.83	493.27

Abbreviations: BR, patients at best response; NA, not available; PD, patients at progression of disease after Ld therapy; pre Ld, patients just before Ld therapy.

^a^ Cytokines included the data outside of the range of the standard curve.

## 4 DISCUSSION

We have shown that higher levels of IL‐18 and M‐CSF were associated with a poor response to Ld therapy and shorter PFS and OS compared to lower levels of these cytokines.

Recently, IL‐18 has been considered as a major factor contributing to the formation of tumor microenvironment. This cytokine promotes MM progression by generating myeloid‐derived suppressor cells (MDSCs) and other immunosuppressive cells and establishing an immunosuppressive milieu. Therefore, high IL‐18 levels in the bone marrow are reported to be an independent poor prognostic factor among patients with MM.^15^ In our study, high IL‐18 levels were associated with poor clinical outcomes to Ld therapy. This is the first report to demonstrate a relationship between IL‐18 and Len efficacy. Several previous reports have shown that Len treatment partially improves the immunosuppressive effect of MDSCs in the tumor milieu.^16^ However, high IL‐18 levels suggest severe immunosuppressive conditions in the tumor milieu that could not be sufficiently improved by Ld treatment. Thus, patients with high IL‐18 levels have poor clinical outcomes to Ld therapy. To overcome the poor outcome in patients with high IL‐18 levels, a triplet regimen of Len contain therapy, such as monoclonal antibody with Ld therapy, may be necessary to improve the severe immunosuppressive conditions. We have shown that high IL‐18 levels were associated with poor response and shorter PFS during Ld therapy. These results suggested the usefulness of IL‐18 as a predictive marker for Ld therapy. Although high IL‐18 levels were also associated with shorter OS, multivariate analysis for OS did not show any independent unfavorable prognostic factors. The future study with large sample size and events is needed to confirm the usefulness of IL‐18 as a prognostic factor.

High M‐CSF levels are predictors of poor survival in patients newly diagnosed with MM.^17^ In our study, the patients with high M‐CSF levels showed poor outcomes after Ld therapy. M‐CSF is involved in osteoclast differentiation and may be associated with excess osteolysis induced by MM progression.^18^ Therefore, Ld therapy may be insufficient for improving the bone environment in patients with high M‐CSF levels. In such cases, the addition of a proteasome inhibitor to Ld therapy may be necessary to improve the bone environment, leading to improved clinical outcomes.

In contrast, higher levels of PDGF‐BB were associated with a favorable response to Ld therapy and were related to longer survival, both PFS and OS, than lower levels among patients with MM treated with Ld therapy. Greco et al. reported that PDGF‐BB can upregulate Myc expression and facilitate Myc‐regulated oncogenic transcription factors in tumor cells.^19^ In MM cells, Myc, and interferon regulatory factor 4 (IRF4) form a positive autoregulatory loop,^20^ and this regulatory loop supports the survival of tumor cells. Thus, high levels of PDGF‐BB may indicate high dependency on Myc‐regulated transcriptional activity in MM cells. Previous studies have shown that Len treatment effectively blocks the positive autoregulatory loop of Myc and IRF4 and decreases survival signaling molecules, such as IKZF1, and IKZF3, in MM cells. Therefore, MM with high PDGF‐BB levels may be highly sensitive to Ld therapy through the effective suppression of Myc‐dependent oncogenic transcriptional activity in MM cells. A decrease of PDGF‐BB levels may suggest that the Myc‐dependent MM clones were eradicated by Ld therapy. We speculate that MM cells proliferated using the alternative pathway instead of the Myc pathway in relapse phase. This might be why PDGF‐BB levels remained low.

In our data, patients with lower G‐CSF, IL‐7, IL‐8, and SDF‐1α levels had shorter OS.

IL‐8 (also known as CXCL8) is a member of the C‐X‐C chemokine family and exhibits angiogenic activity in the bone marrow.^21^ Low levels of SDF‐1α and IL‐8 are associated with the disease progression of MM through migration and homing of MM cells to the bone marrow niche.^22^, ^23^ Pellegrino et al reported that IL‐8 stimulated the proliferation and cell chemotaxis of MM cells. Bone marrow endothelial cells from patients with MM express and secrete higher amounts of IL‐8 than healthy counterpart.^22^ The relationship serum IL‐8 levels in MM patients and Len treatment have not been reported. On the other hand, Zabransky et al reported that Len treatment induced the decrease of IL‐8 levels in non‐progressors of prostate cancer.^24^ We speculate that Len treatment induce the secretion of IL‐8 from bone marrow endothelial cells. Hence, on‐treatment patients might show decreased IL‐8 levels in our study. Moreover, low IL‐8 levels might be associated with the proliferation of MM cells independent IL‐8 and shorter OS.

SDF‐1α and its receptor CXCR4 (C‐X‐C motif chemokine receptor 4) plays a pivotal role in proliferation, invasion, and drug resistance in MM.^25^ SDF‐1α is produced by mainly bone marrow stromal cells and its levels were elevated in BM plasma in patent with MM.^26^ It seemed that high SDF‐1α levels induced the downregulation of CXCR4 and dissemination of MM cells.^23^, ^27^ Alsayed et al. have shown that SDF‐1α levels in the bone marrow of MM patients were higher than those in the peripheral blood.^23^ In our study, OS was significantly shorter in the patients with low SDF‐1α levels in pre‐Ld. This result was not consistent with previous report that have shown SDF‐1α is associated MM progression. We speculate that low SDF‐1α levels in peripheral blood resulted in the upregulation of CXCR4 in surface of MM cells and the formation of EMD. Li et al reported that Len induced upregulation of CXCR4 in CD34^+^ hematopoietic cells.^28^ We speculate that Len reduces the downregulation of CXCR4 on MM cells in bone marrow and prevent the formation of EMD.

In previous report, it was suggested that IL‐7 from MM cells inhibited osteoblast formation and differentiation.^29^ Moreover, IL‐7 also contribute to the increase of osteoclast formation through RANKL stimulation.^30^ Thus, it seems that IL‐7 was associated the bone lesion formation of MM. On the other hand, IL‐7 is essential for the development and survival of T cells.^31^, ^32^, ^33^ IL‐7 was produced by MM cells and bone marrow stroma cells.^32^ In our study, low levels of IL‐7 were associated with poor clinical outcomes of Ld therapy, which may be due to the lower host immune activity of T cells during Len treatment, leading to shorter OS in patients with MM who received Ld therapy. Although it was difficult to detect the source of IL‐7, we thought that decrease of IL‐7 levels after Ld therapy resulted from decrease of MM cells produced IL‐7 and the negative feedback of bone marrow stroma cells produced IL‐7 for T cells proliferation.

Several lines of evidences^9^, ^34^ show that IMiDs modulate the expression of several cytokines in tumor and immune cells in the tumor microenvironment. Notably, IMiDs exert an immune activation effect, such as an immunostimulatory effect on T cells and NK cells, and an inhibitory effect on tumor growth by interfering with tumor microenvironment interactions, such as anti‐angiogenesis.^35^

Schütt et al. reported that Thal increased serum soluble IL‐2R via NK cell activation.^36^ Other previous report suggested that serum sIL‐2R did not increase in PD.^3^ Therefore, it might be difficult to use sIL‐2R as tumor marker especially during IMiDs containing regimen. It seems that decreased NK cell activity might be due to relapse of MM and sIL‐2R decrease. sIL‐2R might be affected the tumor volume and NK cell activity and not be increased in relapse.

In our cohort, the level of IFN‐γ, one of the Th1 cytokines, was elevated during Ld therapy, and this elevation disappeared during disease progression. This result indicated that the activation of T cells was triggered during Len treatment, and this activation was canceled by the growth of Len‐resistant MM cells. In previous report, serum IFNγ levels were lower in MGUS and MM than healthy control.^3^ We thought that serum IFNγ levels in the patients with MM are permanently lower than healthy individuals. Therefore, it is needed that the comparison between low levels of IFNγ in patients with RRMM. In our study, it seemed that the secretion of IFNγ were slightly recovered in best response to Ld therapy (Figure 4D).

TNF‐α, a major cytokine playing a critical role in tumor microenvironment interactions, is decreased during Ld therapy, and this level did not change with disease progression. With Len treatment, tumor microenvironment interactions supported by the transfer of inflammatory cytokines becomes invalid by the modulation of cytokine levels in the microenvironment, represented by the reduction of TNF‐α level. Even during disease progression, TNF‐α level was low. This result suggests that specific MM clones with low microenvironment dependency are refractory to Len treatment and proliferate without tumor microenvironment support.

Portier et al. reported that G‐CSF gene was expressed in most patients with MM.^37^ Xu et al. suggested that G‐CSF induces the production of TNF‐α.^38^ In our study, the positive correlation between G‐CSF levels and TNF‐α levels was shown (Figure S6). This was consistent with previous report. We speculate that high G‐CSF levels associated with high TNF‐α levels in serum. Len induces the reduction of TNF‐α level and may also induce the reduction of G‐CSF. Thus, the patients with low G‐CSF levels in pre‐Ld might be associated with poor outcome of Ld therapy because of low levels of TNF‐α.

The expression levels of IL‐4 and IL‐6, an inflammatory and Th2 type cytokine, respectively, were low during Len treatment, indicating the inhibitory effects of Len on inflammatory and Th2‐type cytokines. However, during the progression stage, the levels of these cytokines increased. Kyrstsonis et al. reported a correlation between two cytokines, IL‐4 and IL‐6, and disease progression and prognosis.^39^ Therefore, elevated levels of these cytokines may be related to the growth of Len‐resistant MM clones. In our cohort, 82% of the patients in pre‐Ld group had previously received bortezomib‐containing therapies. Cytokine levels of IL‐4 were lower in the patients who had prior Bor therapy (Figure S3). Low levels of IL‐4 in pre‐Ld might be influenced by prior Bor therapy. In previous report, serum IL‐6 levels ranged from 0 to 13 pg/mL (median 5 pg/mL).^39^ In addition, although an elevation of IL‐6 in the bone marrow plasma was observed, IL‐6 levels in peripheral blood plasma were not elevated in previous study.^3^ Our results were consistent with these previous reports. It seems that IL‐6 levels tended to be low in peripheral blood and were compared within the range of low values.

Our study had some limitations. First, further validations on the other cohorts are needed to evaluate our results. Second, although all samples were collected just before Ld therapy, no samples were collected at the time of diagnosis or before the initial therapy. Thus, no comparison of the samples between the initial therapy and Ld therapy could be performed. Third, the background of prior therapy in patients with MM was not uniform in our cohort. Therefore, further studies are required to identify the role of cytokines as predictive biomarkers of Ld therapy efficacy. Fourth, it is difficult to evaluate the tumor microenvironment using serum cytokines alone. Cytokines might be secreted from not only myeloma cells but also lymphocyte, other immune cell and stromal cells. However, cytokine levels from each cell and CD4/CD8 ratio were not analyzed in this study. Moreover, the cytokine concentrations in bone marrow and peripheral blood may diverge, and that cytokine levels may change depending on the timing of blood collection, gender, and age.

In conclusion, we demonstrated that high IL‐18 and M‐CSF levels and low PDGF‐BB levels are associated with poor clinical outcomes to Ld therapy. Moreover, low G‐CSF, IL‐7, IL‐8, and SDF‐1α levels were associated with shorter OS after Ld therapy. This study provides a better understanding of the association between cytokines and Ld therapy efficacy. Our results may contribute to the identification of a specific patient population who might benefit from Len‐containing therapy.

## AUTHOR CONTRIBUTIONS

**Takuto Tachita:** Conceptualization (equal); data curation (lead); formal analysis (lead); investigation (lead); methodology (equal); project administration (equal); software (equal); visualization (lead); writing – original draft (lead); writing – review and editing (lead). **Masaki Ri:** Conceptualization (equal); formal analysis (supporting); investigation (supporting); methodology (equal); project administration (equal); resources (lead); software (supporting); supervision (equal); visualization (supporting); writing – original draft (supporting); writing – review and editing (lead). **Sho Aoki:** Writing – review and editing (supporting). **Arisa Asano:** Writing – review and editing (supporting). **Takashi Kanamori:** Writing – review and editing (supporting). **Haruhito Totani:** Writing – review and editing (supporting). **Shiori Kinoshita:** Writing – review and editing (supporting). **Yu Asao:** Writing – review and editing (supporting). **Tomoko Narita:** Writing – review and editing (supporting). **Ayako Masaki:** Writing – review and editing (supporting). **Asahi Ito:** Writing – review and editing (supporting). **Shigeru Kusumoto:** Writing – review and editing (supporting). **Hirokazu Komatsu:** Writing – review and editing (supporting). **Shinsuke Iida:** Project administration (supporting); resources (equal); supervision (equal); writing – review and editing (supporting).

## FUNDING INFORMATION

This work was partly supported by a Grant‐in‐Aid for Scientific Research from the Ministry of Education, Culture, Sports, Science, and Technology (16K07179 & 16K09855), the National Cancer Center Research and Development Fund (26‐A‐4), and Practical Research for Innovative Cancer Control from the Japan Agency for Medical Research and Development, AMED (15ck0106077h0002).

## CONFLICT OF INTEREST STATEMENT

MR received research funding from Celgene Co., Ltd. SI received research funding and declared Honoraria from Janssen Pharmaceutical K.K., and Celgene Co., Ltd. SI also received research funding from Kyowa Hakko Kirin Co. Ltd., Chugai Pharmaceutical Co. Ltd., Bristol‐Myers Squibb, Ono Pharmaceutical Co. Ltd., and Nippon Kayaku Co. Ltd. Eli Lilly Japan K.K. and Bayer Yakuhin Ltd. Other authors do not have COI.

## ETHICS STATEMENT

*Approval of the research protocol by an Institutional Reviewer Board*: This study was approved by the Ethical Committee of Nagoya City University Graduate School of Medical Sciences (ID: 70‐00‐0113).

*Informed consent*: All patients provided written informed consent prior to peripheral blood sampling according to the requirements of the Declaration of Helsinki.

*Registry and the registration no. of the study/trial*: N/A

*Animal studies*: N/A

## Supporting information


Figure S1.

Figure S2.

Figure S3.

Figure S4.

Figure S5.

Figure S6.

Table S1.

Table S2.

Table S3.

Table S4.

Table S5.

## Data Availability

The data that support the findings of this study are available from the corresponding author (MR) upon reasonable request.
