# Supplementary figures and images for: Comprehensive analysis of serum cytokines in patients with multiple myeloma before and after lenalidomide and dexamethasone
Source: Cancer Med. 2024 Jul 19;13(14):e70019. doi: 10.1002/cam4.70019 (PMC11259000; doi:10.1002/cam4.70019)

Supplementary Figure 1.

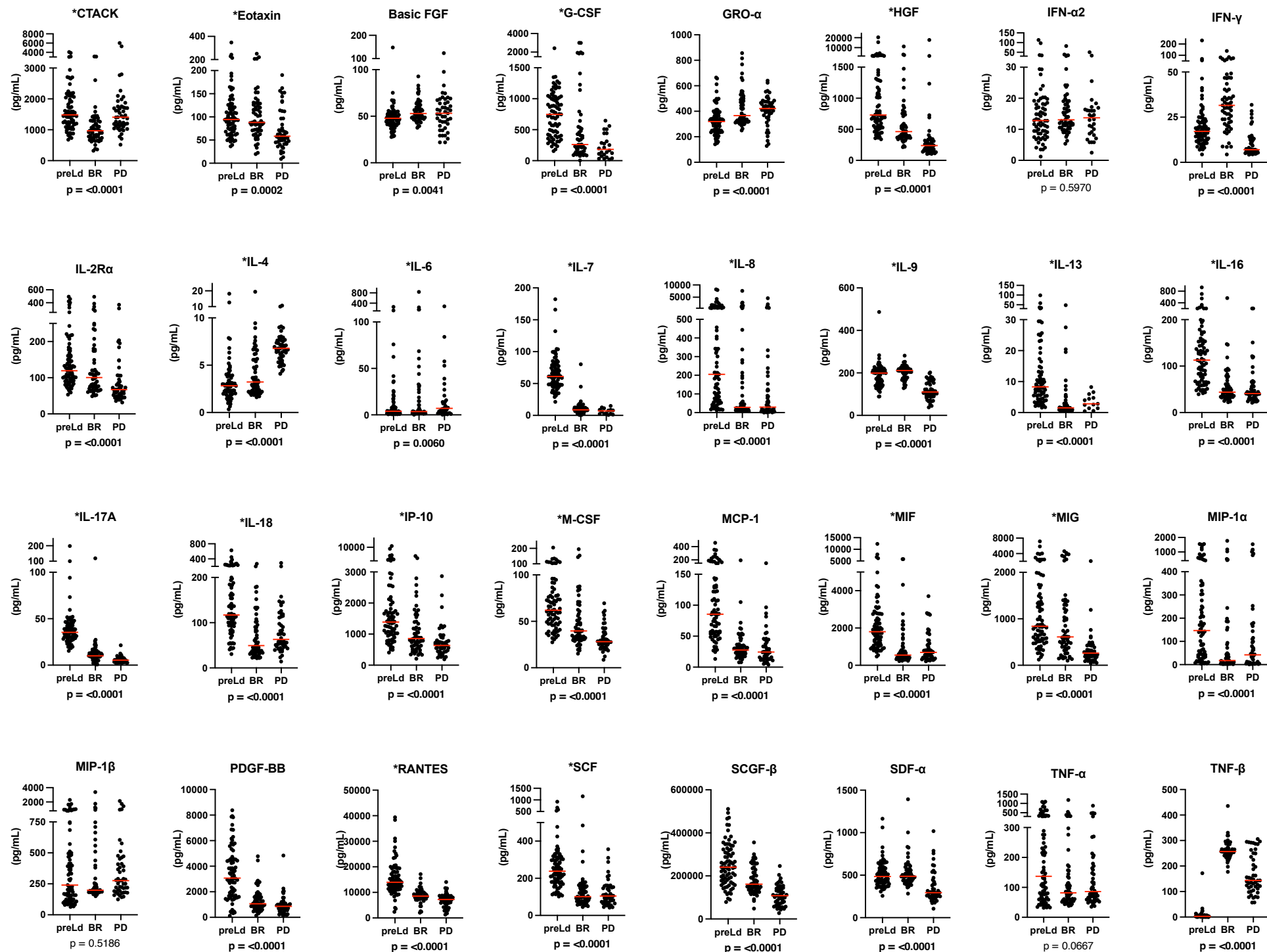

Supplement: Supplementary file 1 — Figure S1. [file CAM4-13-e70019-s006.pdf]

Supplementary Figure 2.

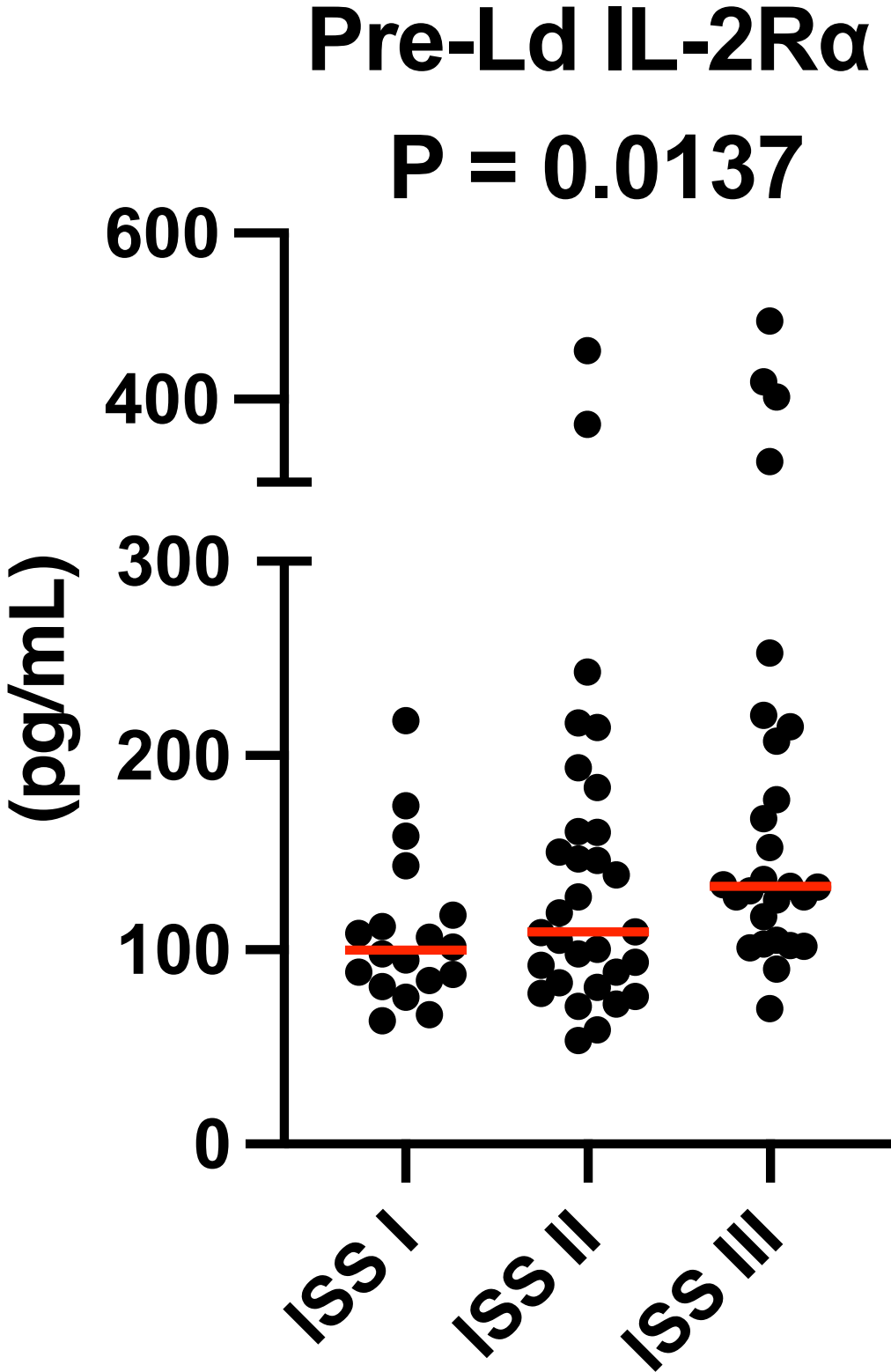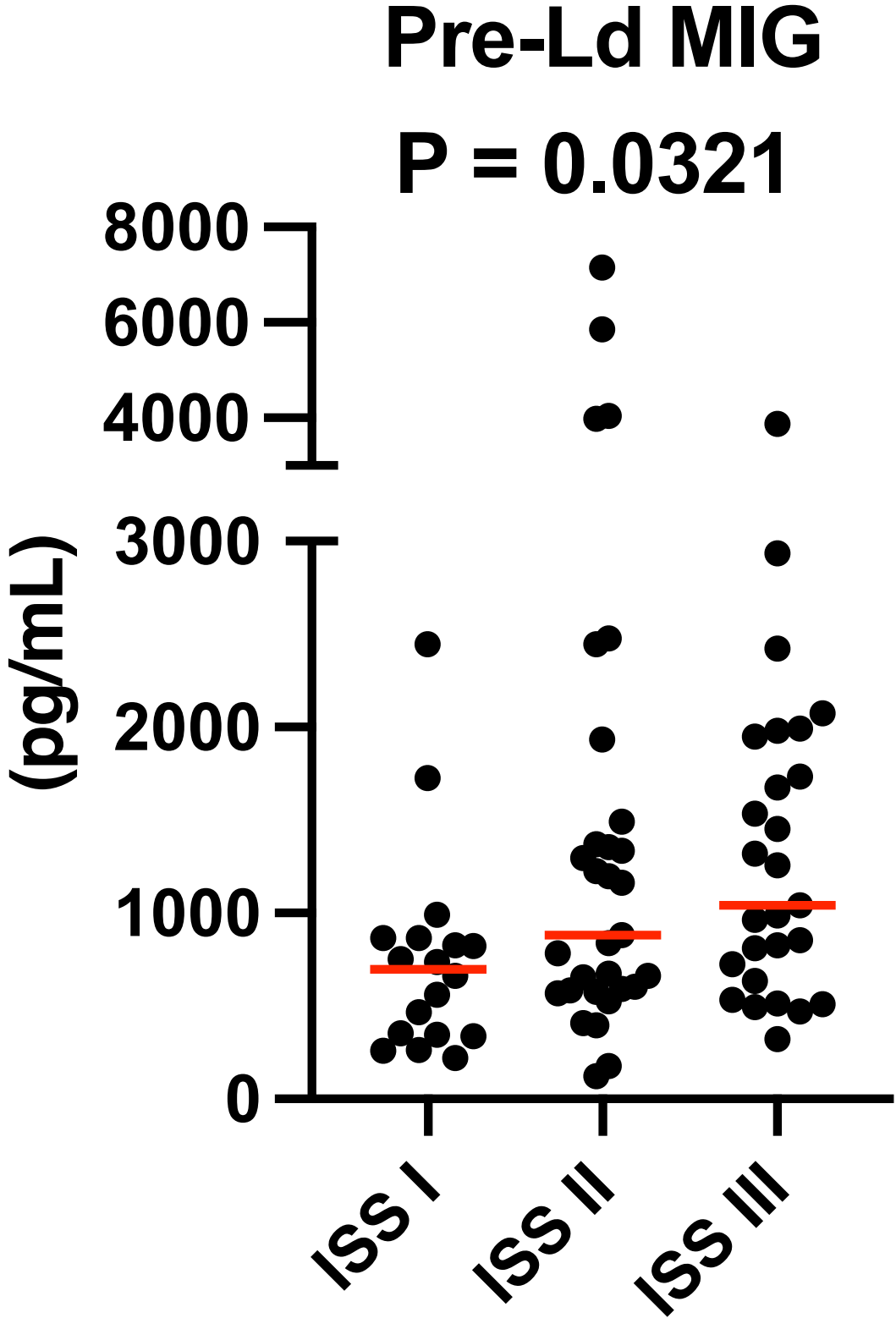

Supplement: Supplementary file 2 — Figure S2. [file CAM4-13-e70019-s011.pdf]

Supplementary Figure 3.

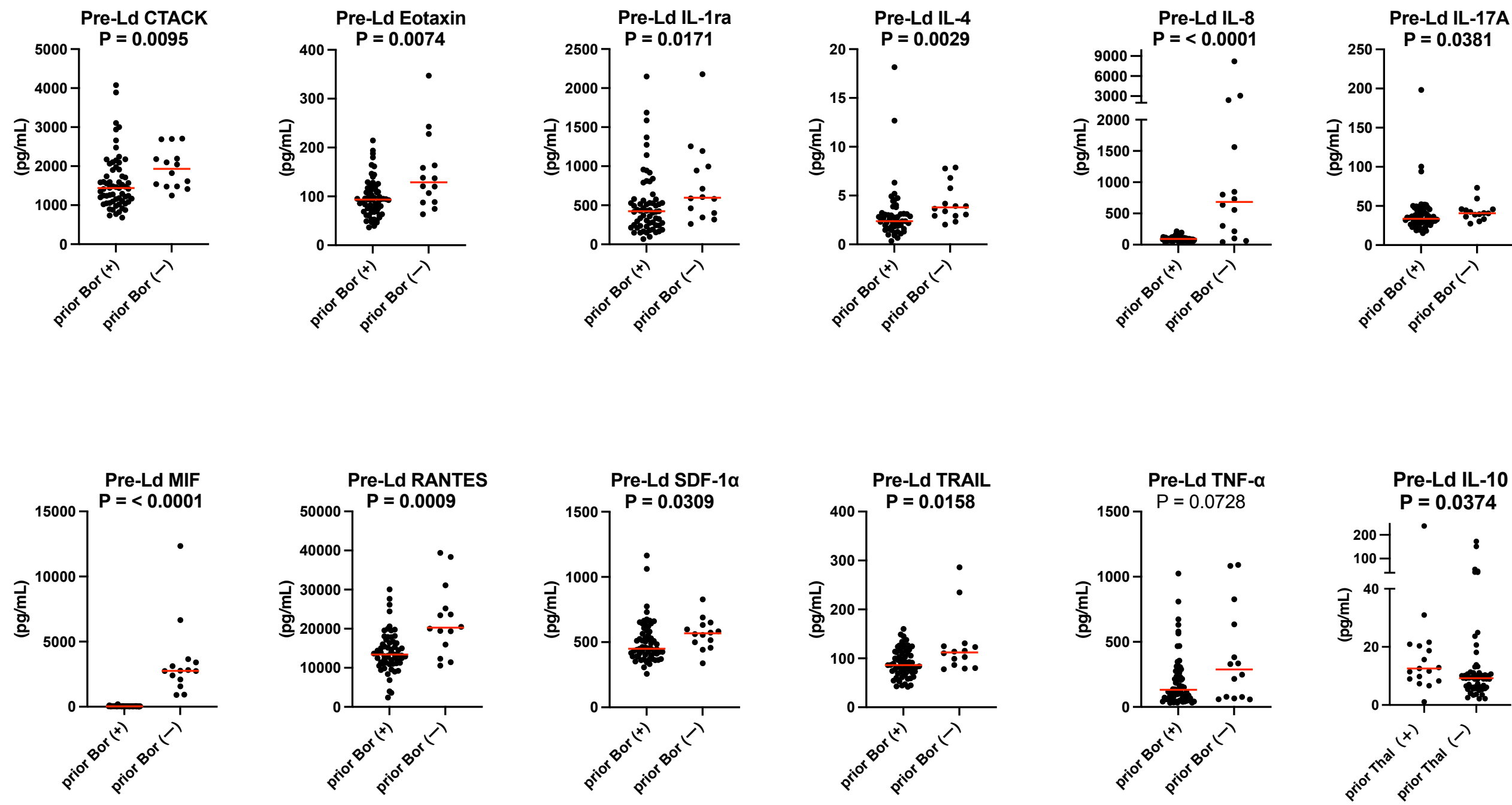

Supplement: Supplementary file 3 — Figure S3. [file CAM4-13-e70019-s001.pdf]

Supplementary Figure 4.

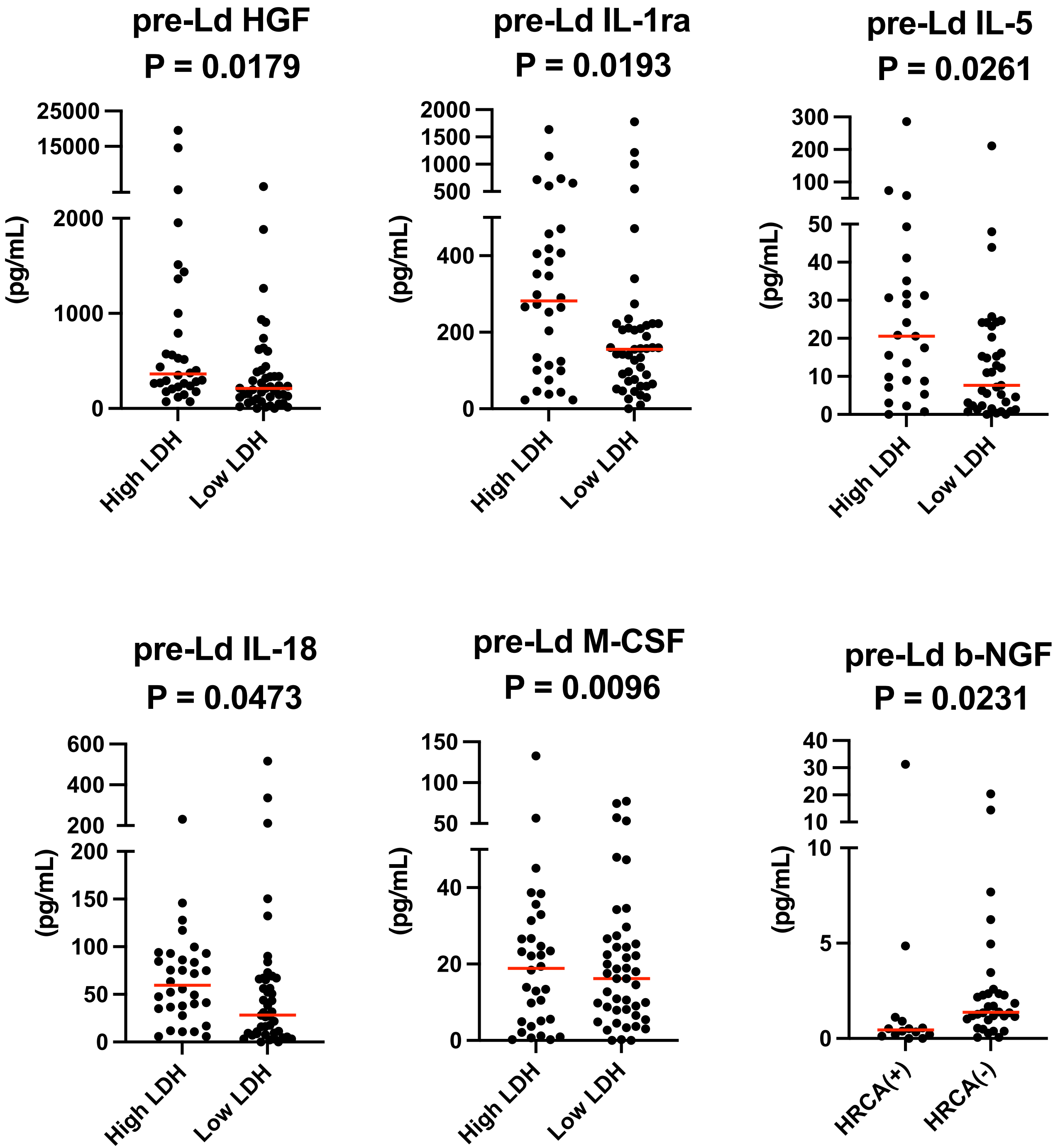

Supplement: Supplementary file 4 — Figure S4. [file CAM4-13-e70019-s009.pdf]

**Supplementary Figure 5.**

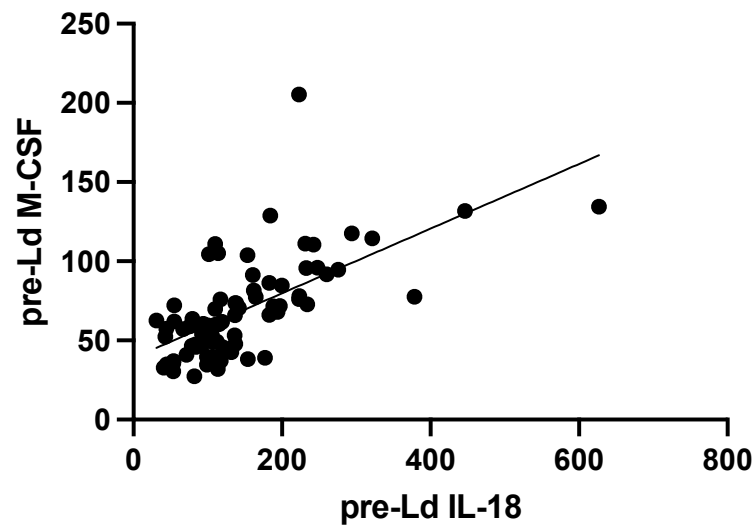

**P = <0.0001**  
**R<sup>2</sup> = 0.4167**

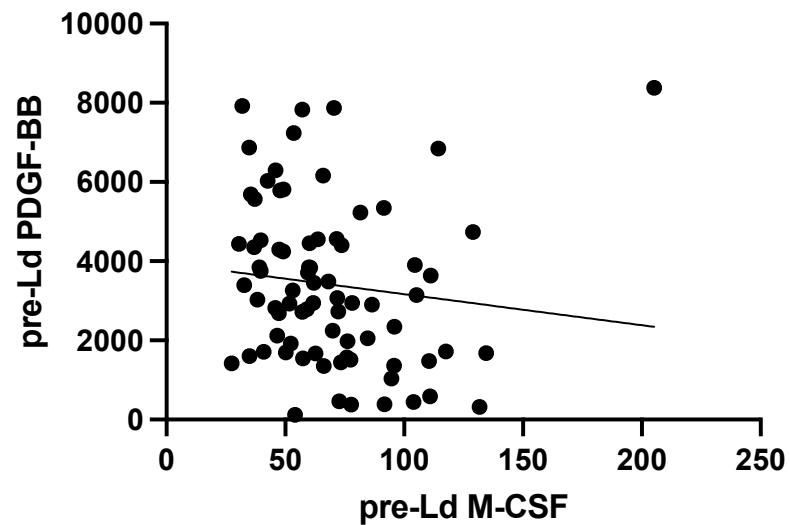

**P = 0.3116**  
**R<sup>2</sup> = 0.01365**

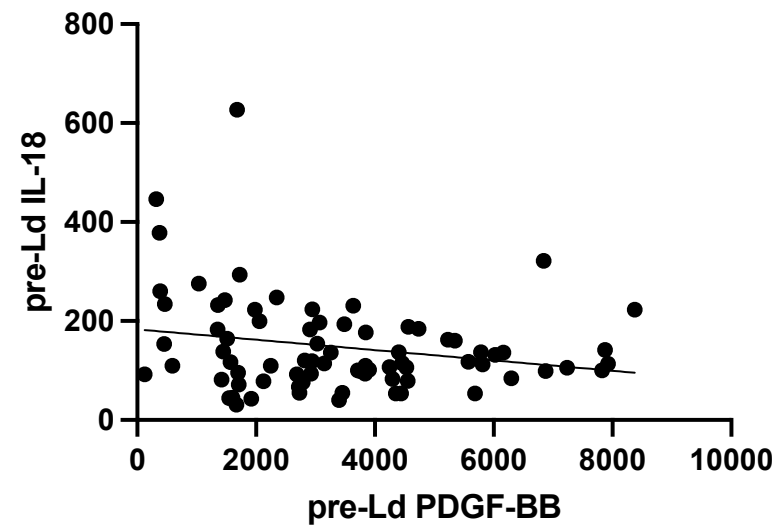

**P = 0.0529**  
**R<sup>2</sup> = 0.04903**

Supplement: Supplementary file 5 — Figure S5. [file CAM4-13-e70019-s005.pdf]

Supplementary Figure 6.

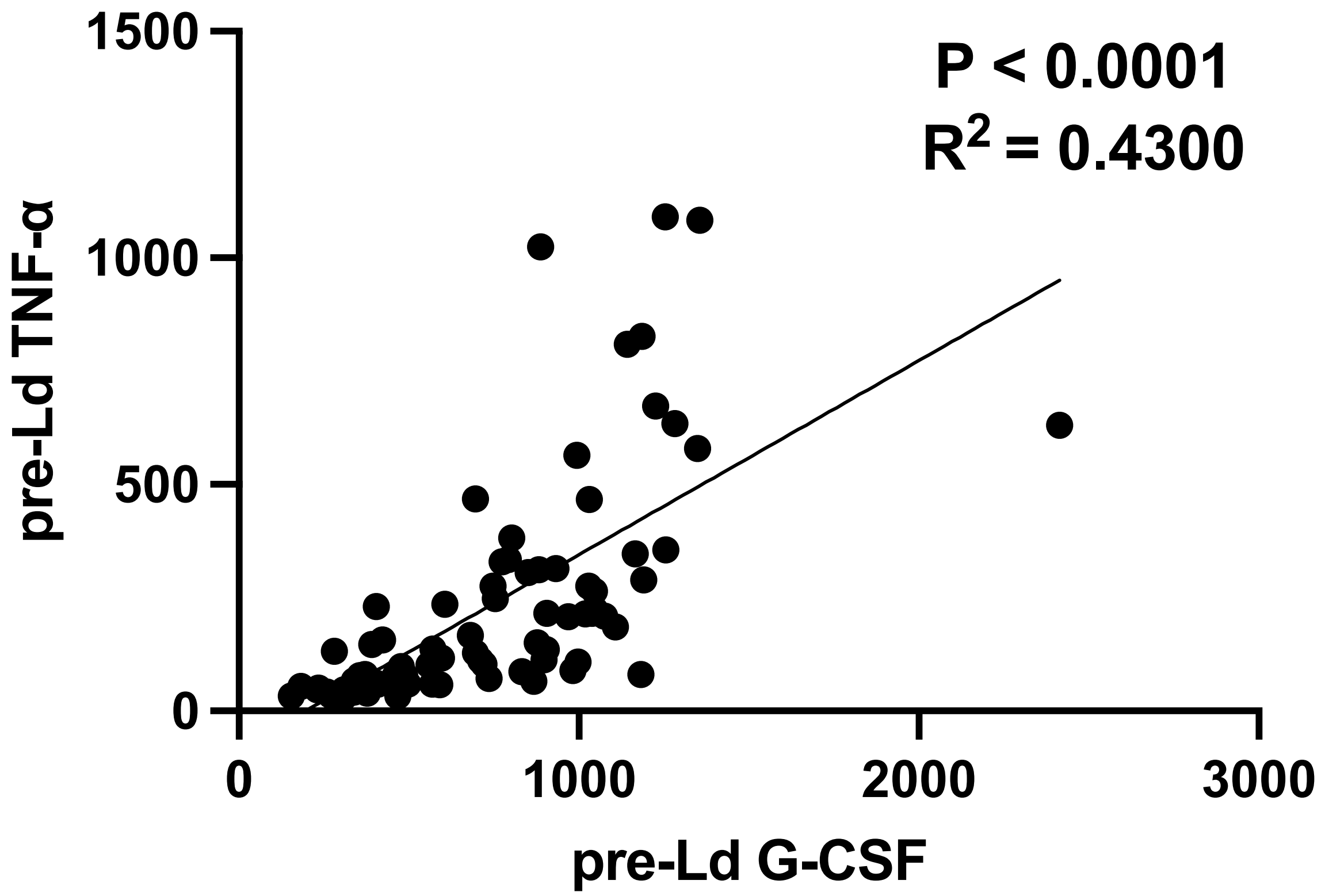

Supplement: Supplementary file 6 — Figure S6. [file CAM4-13-e70019-s004.pdf]
